# Post-ivermectin encephalopathy in Senegal: a case report

**DOI:** 10.11604/pamj.2017.27.202.12106

**Published:** 2017-07-18

**Authors:** Daniel Gams Massi, Mohamed Lelouma Mansare, Mariétou Traoré, Moustapha Ndiaye, Amadou Gallo Diop, Mouhamadou Mansour Ndiaye

**Affiliations:** 1 Cheikh Anta Diop University, Neurosciences Department, Fann National Teaching Hospital, Dakar, Senegal

**Keywords:** Ivermectin, encephalopathy, corticosteroids

## Abstract

Ivermectin is an ant parasitic drug used for combating onchocerciasis and lymphatic filariasis. It works by inhibiting the function of neurons and muscles, thus causing paralysis of microfilariae. Side effects of this drug have been reported including post-ivermectin encephalopathy requiring emergency care in hospital. We report the case of a 35 years old patient living in rural areas of Senegal who presented two days after a mistake in administration of a second dose of ivermectin, headaches, altered consciousness and bilateral blindness. The workup revealed brain white matter lesions, abnormal liver function tests and biological inflammation without evidence of Loa loa microfilariae in blood and cerebrospinal fluid. Corticosteroid treatment was administered in emergency and patient recovered despite the persistence of bilateral blindness. Inflammatory process seems to have an important role in the pathophysiology of this encephalopathy. We should therefore carefully control the administration of this drugs.

## Introduction

Ivermectin is an antiparasitic drug derived from Avermectin massively used in the fight against onchocerciasis and lymphatic filariasis in Africa and Latin America [1]. Its mechanism of action is partly linked to the slow and irreversible opening of glutamate-gated chloride channels (GluCl) responsible for prolonged hyperpolarization or depolarization of muscle cells and neurons [2]. Several cases of post-ivermectin encephalopathy have been reported in the literature as “Probable Loa encephalopathy temporally related to ivermectin” (PLERI) mainly in Democratic Republic of Congo and Cameroon [2–5]. This definition is related to the onset of neurological signs with evidence of Loa loa microfilariae in the blood and/or cerebrospinal fluid (CSF) after administration of ivermectin [6]. We report a case of encephalopathy occurred in Senegal after administration of ivermectin without evidence of microfilariae in the blood.

## Patient and observation

**Figure 1:**
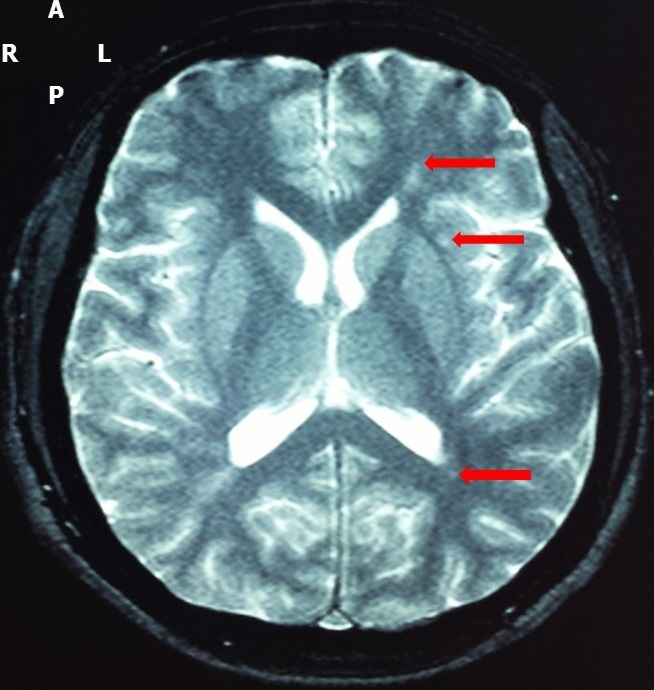
Axial T2-weighted brain MRI showing (in red arrows) hypersignal lesions located in the left semi-oval center and periventricular area: A = anterior; P = posterior; L = left; R = right

**Figure 2:**
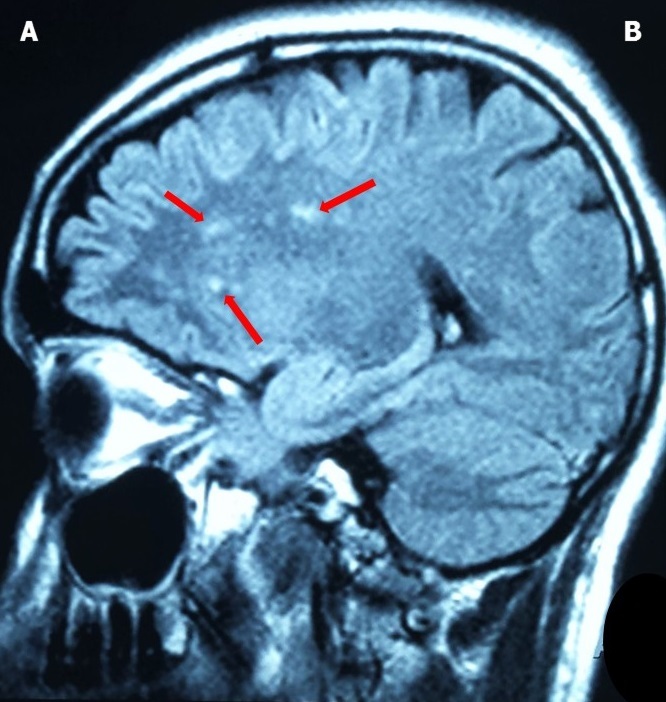
Sagittal FLAIR brain MRI showing (in red arrows) hypersignal lesions located in the left semi-oval center: A = anterior; P = posterior

He is a 35 years old patient living in Mbirkilane (Kaffrine region of Senegal) where he grew up, with no past medical, no travel history nor onchocerciasis. He was admitted in the neurology department of Fann national teaching hospital on May 26, 2016. Patient received two doses of ivermectin (150 mcg/kg body weight). Ivermectin was given on May 9^th^ and May 24^th^, 2016 for the first and second dose respectively. This second dose was due to a mistake of patient who did not advised the drug's distributor about the previous dose of ivermectin. Two days after receiving this second dose, he presented bilateral eye pains, blurred vision and moderate to severe headaches associated to vomiting, followed few hours later by generalized tonic-clonic seizures. He was first admitted and treated in the regional hospital without improvement. The onset of altered consciousness motivated the transfer in our neurology department.The clinical examination revealed headaches, tonic-clonic seizures, obtundation (Glasgow coma score of 10/15), bilateral visual loss and unreactive mydriasis, bradycardia (51 beats per minute) and a weight of 68 kilograms. The funduscopy found bilateral papilledema with retinal hemorrhages. The patient was urgently admitted in the intensive care unit of the department. Neuroimaging done showed multiple hypersignal lesions in T2 weighted and FLAIR (fluid-attenuated inversion recovery) brain MRI, located in the periventricular white matter and semi-oval centers (Figure 1, Figure 2). The visual evoked potentials found bilateral signs of optic nerve's axonal loss and demyelination.

The results of cerebrospinal fluid analysis were: albumin 0.49 g / L (normal < 0.5 g/L), glucose 0.69 g/L (normal ≥ half of concomitant fasting blood sugar that was 1.22 g/L), white blood cells < 2 cells/mm^3^ (normal < 5 cells/mm^3^), the research of bacteria, virus, fungi and parasites remained negative. The blood sample was collected in the morning of May 27^th^, 2016 for the research of microfilariae and dosage of anti-Acanthocheilonema vitae Immunoglobuline G and the results were negatives. The liver function test was impaired with SGOT at 396.8 IU / L (normal <40 IU/L), SGPT at 95,5UI/L (normal <41 IU/L) and Gamma-Glutamyltransferase at 72 IU/L (normal: 12-64 IU/L). The HBs antigen, anti-HCV antibodies and the alpha-fetoprotein remained negatives and abdominal ultrasound normal. We found all possible causes of encephalopathy in our context. CRP was 14.74 mg/l which is slightly increased and leukocytosis at 14 660/mm^3^(normal: 4000-10000/mm^3^) predominantly neutrophilic with no isolated germ and no Strongyloides were isolated in this patient. Diagnosis of post-ivermectin encephalopathy was retained and the patient was treated with corticosteroids: Betamethasone 0.1 mg/kg/day then Prednisone 1mg/kg/day associated to calcium and potassium supplementation, proton pump inhibitor (Omeprazole 20 mg/day), analgesics and hydration with normal saline.The evolution was marked by the return to consciousness two days after admission, the withdrawal of headaches, vomiting and seizures during the first week of hospitalization. But the bilateral blindness still persists until day despite an appropriate ophthalmologic treatment. The patient has been discharged from the hospital on July 22, 2016 and is currently followed in collaboration with ophthalmologists. The patient agreed an inform consent form giving us his approval to publish a case report.

## Discussion

This encephalopathy was described mainly in adult males living in rural areas [4–6]. Why this severe side effect appears only in males remain unexplained. It seems important to consider the presence of protective factors in women, especially hormones. This preventive strategy initiated in 2000, has contributed to the reduction of infectivity rate of Onchocerca Volvulus to about 0‰ in Senegal according to Diawara et al [7]. By mistake, our patient received two doses of ivermectin 150 mcg/kg in fifteen days interval, which is not recommended by the world health organization guidelines [8]. Clinical manifestations often include altered consciousness, gait and speech disturbances, headaches, back pain, urinary incontinence and visual loss [3, 4, 6]. The slightly elevated CRP might be due to the fact that the patient was previously admitted in a regional hospital where he probably received anti-inflammatory drugs as usual in peripheral health centers of Senegal. In most cases, the presence of Loa loa microfilariae has been demonstrated in the peripheral blood or CSF before and after treatment with ivermectin [6]. Despite extensive research, we have not found Loa loa microfilariae in peripheral blood and CSF suggesting that ivermectin itself may induce encephalopathy. Some studies reported on post-mortem brain slides perivascular inflammatory infiltrates with hemosiderin deposits, thickening of the basal membrane with rupture of the vascular wall, a reactive gliosis with moderate demyelinating lesions [3, 4]. The MRI lesions in the brain white matter may correspond to the inflammatory lesions found in neuropathology. The evolution is not really spectacular and sometime requires intensive care and corticosteroid [3–6].

## Conclusion

Encephalopathy after administration of ivermectin is a severe and rare complication of this treatment especially in case of presence of Loa loa microfilariae. Neurologic complications appears rarely in the absence of microfilariae. The inflammation of white matter in the brain may contribute to the severity of this disease. We should be more careful regarding the community-based administration of ivermectin.

## Competing interests

The authors declare no competing interest.
